# Enhanced visible/near-infrared spectroscopic data for prediction of quality attributes in Cucurbitaceae commodities

**DOI:** 10.1016/j.dib.2021.107458

**Published:** 2021-10-08

**Authors:** Kusumiyati Kusumiyati, Yuda Hadiwijaya, Ine Elisa Putri, Agus Arip Munawar

**Affiliations:** a Department of Agronomy, Faculty of Agriculture, Universitas Padjadjaran, Sumedang 45363, Indonesia; b Department of Agricultural Engineering, Faculty of Agriculture, Universitas Syiah Kuala, Banda Aceh 23111, Indonesia

**Keywords:** Calibration model, Post-harvest, Prediction, Spectral data, Spectroscopy, Technology

## Abstract

Spectra data of 300 samples from 6 Cucurbitaceae commodities, including zucchini, bitter gourd, ridge gourd, melon, chayote, and cucumber, were recorded using a handheld visible/near-infrared (Vis/NIR) instrument. Vis/NIR data were obtained in the form of absorbance spectra data at a wavelength of 381–1065 nm. The spectral data has the potential to be reused to predict quality attributes in the form of soluble solids and water content on several Cucurbitaceae commodities. The accuracy of the Vis/NIR calibration model can be increased by applying spectra preprocessing, for example, second derivative savitzky-golay (dg2). The calibration model was developed using the principal component regression (PCR) method on RAW and dg2 spectra. The enhanced Vis/NIR dataset can be used to evaluate the inner quality attributes of intact fruits in a rapid, non-destructive manner.

## Specifications Table

**Table:** 

Subject	Agricultural Sciences Post-harvest Technology
Specific subject area	Spectroscopy, non-destructive technique in agriculture.
Type of data	Table Graph Spectroscopic data
How data were acquired	Spectra datasets were acquired using NirVana AG410 (Integrated Spectronics Pty, Ltd, Australia) spectrometer. The wavelength range used was 381-1065 nm, and the data were reported as absorbance.
Data format	Raw Analyzed Enhanced Presented as .xls and .unsb file formats
Parameters for data collection	Data were collected from a total of 300 samples consisting of 6 commodities in the Cucurbitaceae family.
Description of data collection	The visible/near-infrared dataset is obtained from 300 samples. Each sample was scanned six times and averaged to acquire a spectrum for each sample. Reference data for the quality parameters of soluble solids and water content were carried out by standard laboratory methods. The measurement of soluble solids content was carried out using the refractive index method using a refractometer, while the determination of the water content was carried out by the drying method using an oven at 60 °C.
Data source location	Data were collected at the Laboratory of Horticulture, Department of Agronomy, Faculty of Agriculture, Universitas Padjadjaran, Sumedang, Indonesia
Data accessibility	The combined dataset are provided as Microsoft Excel (.xlsx) and The Unscrambler (.unsb) extension formats. The dataset is available on this article and can be found in Mendeley repository data: https://data.mendeley.com/datasets/k55b8mvs84/1
Related research article	Kusumiyati, Hadiwijaya, Y., Putri, I. E., & Munawar, A. A. (2021). Multi-product calibration model for soluble solids and water content quantification in Cucurbitaceae family, using visible/near-infrared spectroscopy. *Heliyon, 7*(8). https://doi.org/10.1016/j.heliyon.2021.e07677

## Value of the Data

• Spectra data set on fresh samples of 6 commodities, including zucchini, bitter gourd, ridge gourd, melon, chayote, and cucumber, to predict soluble solids and water content non-destructively.
• Data were beneficial in agricultural industries for quality inspection, sorting, and grading.
• The calibration model developed from the dataset can be transferred to the Vis/NIR instrument.
• The dataset can also be remodeled through different spectra preprocessing and regression approaches to enhance prediction accuracy.
• Combining enhanced spectral data with an appropriate regression approach would result in a more reliable calibration model.

## 1 Data Description

The quality of food is essential to take into account because it impacts the consumer acceptance. Therefore, agricultural commodities need to be ensured in a good physical and chemical condition. The destructive method is commonly used to determine the quality of fruit, such as their soluble solids and water content. Visible/near-infrared (Vis/NIR) spectroscopy is an interesting study to research because of its many benefits, especially in agriculture. Vis/NIR works based on the interaction between biological objects and electromagnetic waves in the visible and near-infrared regions. Vis/NIR spectra data can be used to determine various quality parameters in agricultural commodities such as fruits, vegetables, and others [1]. The peak at a particular wavelength indicates that the wavelength has a significant influence in predicting the chemical content of the sample [2]. This technique has several advantages, including predicting the quality of agricultural commodities quickly, accurately, and nondestructively.

**Fig. 1:**
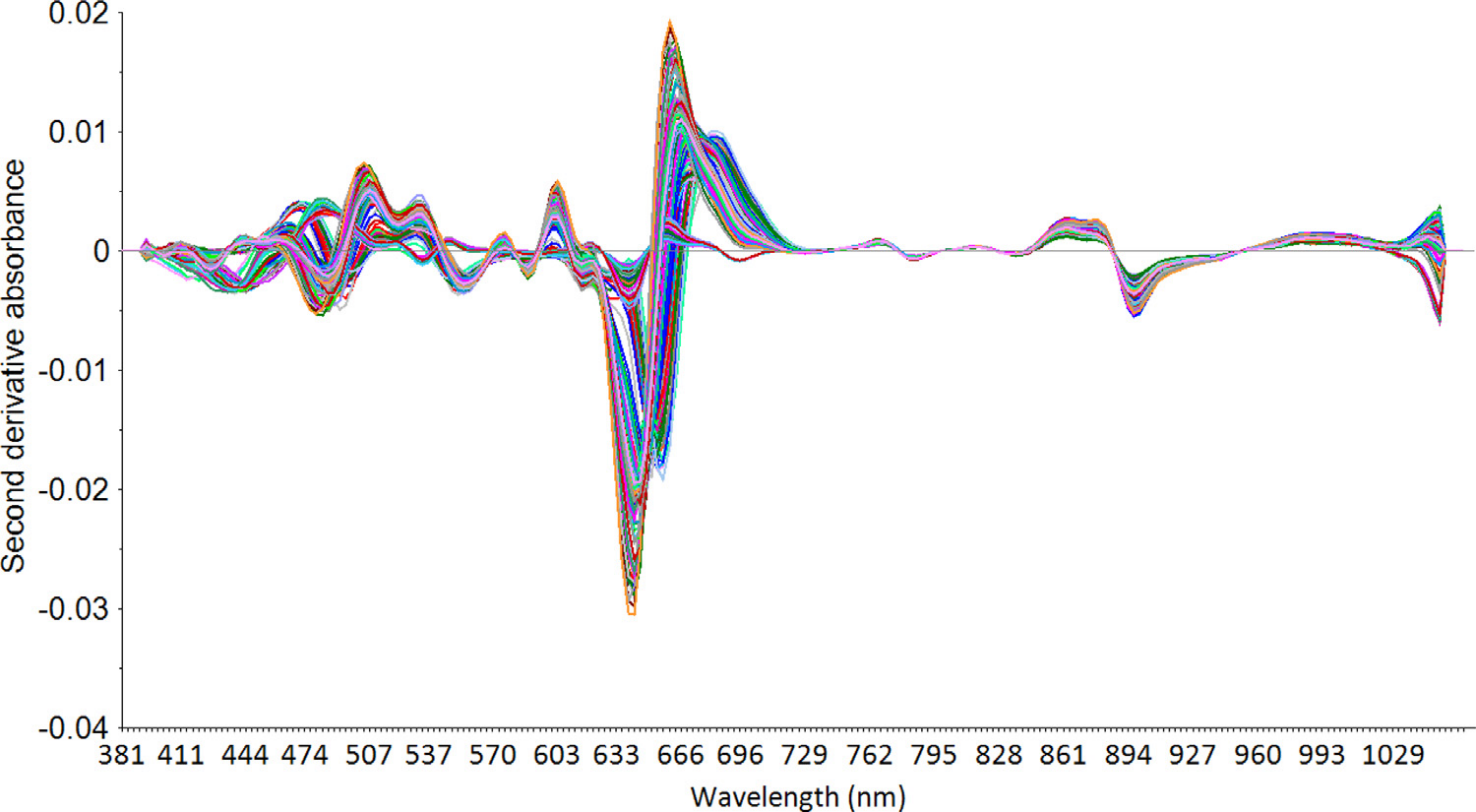
Second derivative savitzky-golay spectra.

Visible/near-infrared spectra data were obtained in the form of absorbance spectra at a wavelength of 381–1065 nm. The spectra data were then enhanced by applying the spectra preprocessing to the original spectra. The purpose of the spectra preprocessing is to improve the accuracy of the calibration model. One of the most frequently used techniques is the second derivative savitzky-golay (dg2) (Fig. 1).

**Fig. 2:**
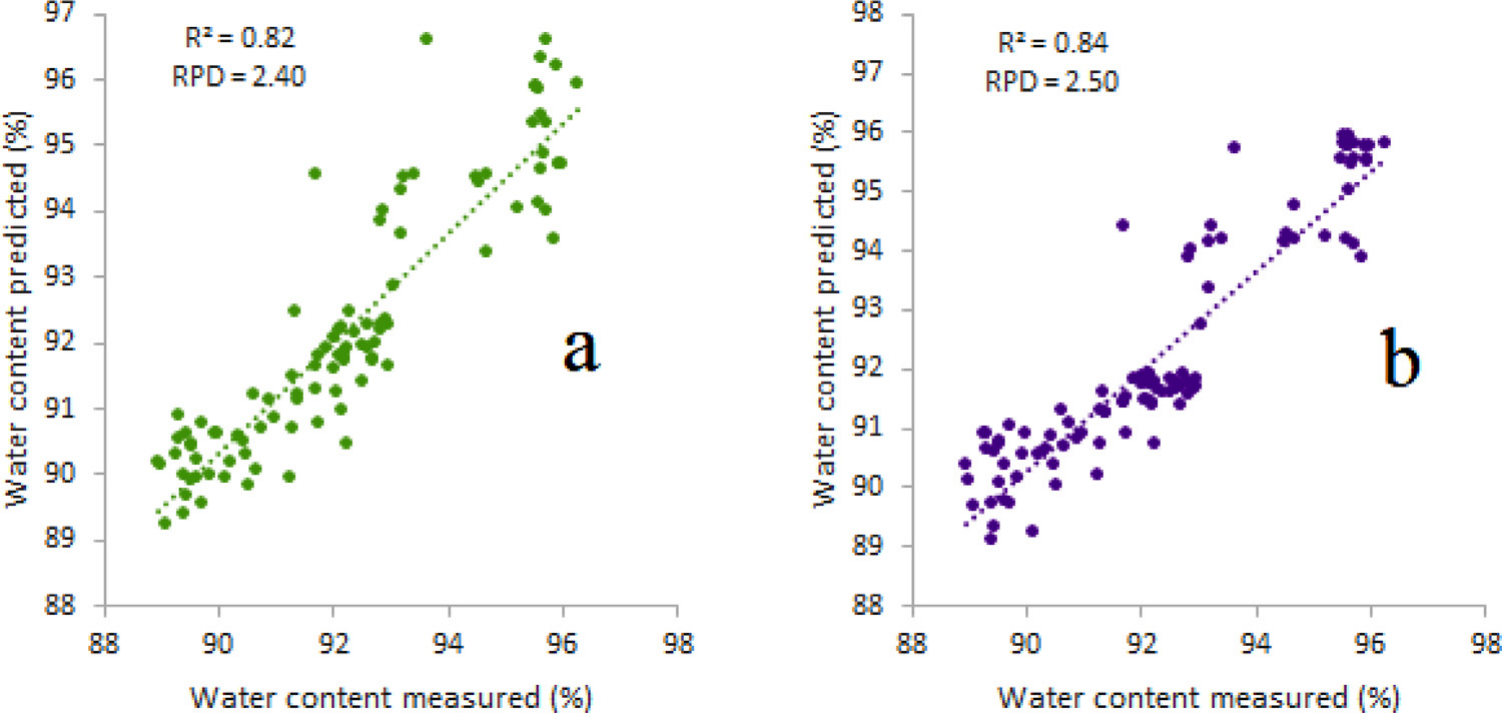
Prediction performance on raw (a) and dg2 (b) spectra in predicting water content in Cucurbitaceae commodities.

**Fig. 3:**
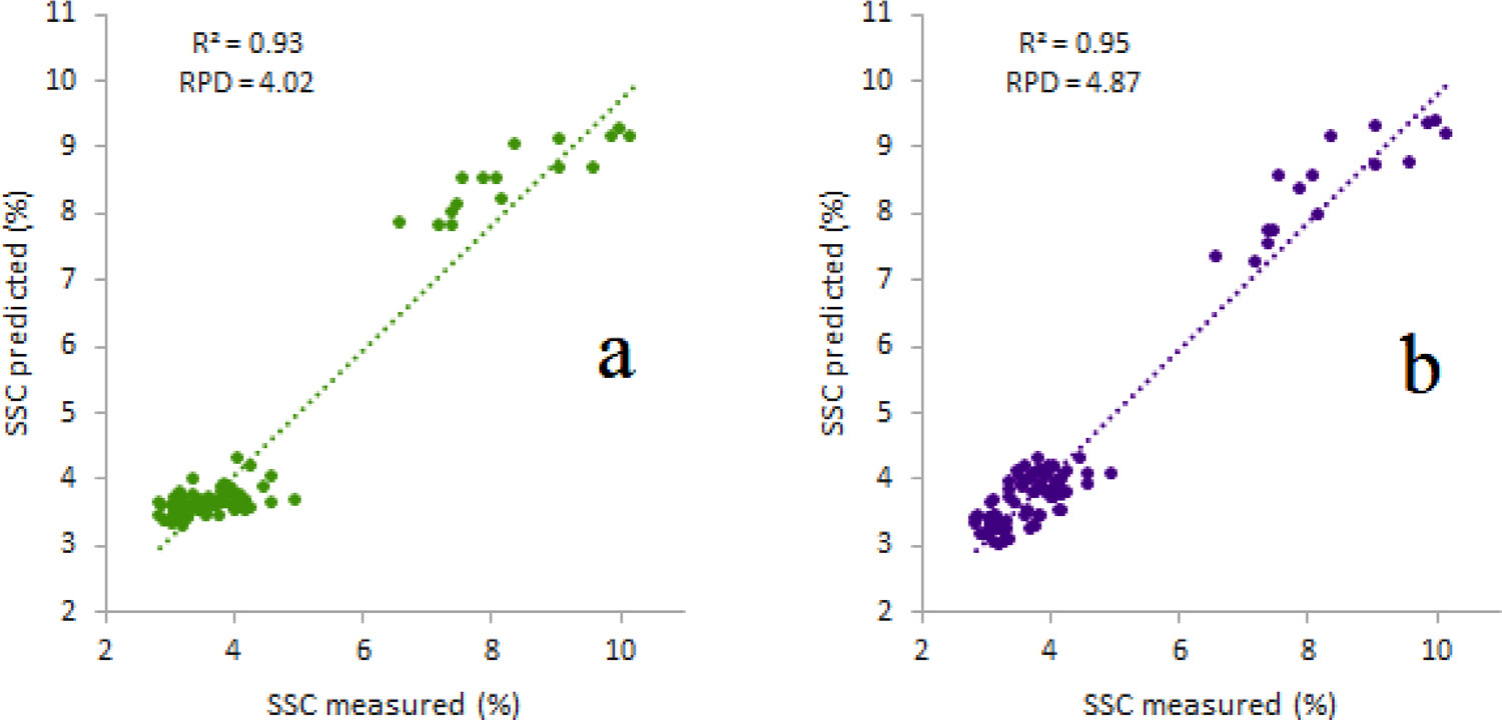
Prediction performance on raw (a) and dg2 (b) spectra in predicting soluble solids content (SSC) in Cucurbitaceae commodities.

The Vis/NIR calibration model can be developed using either untreated or enhanced spectra. Table 1 displays the predictive performance of Vis/NIR for quantify water content and SSC. Figs. 2 and 3 display a scatter plot of the predicted results on both types of spectra and quality parameters. The application of preprocessing spectra showed an increase in the prediction accuracy of both quality attributes.

**Table 1:** Comparison of model performance between raw and enhanced spectra data using principal component regression (PCR) algorithm.

	Statistical indicators				
Quality attributes	Spectra data	R^2^	r	RMSE	RPD
Water content	RAW	0.82	0.90	0.89	2.40
	dg2	0.84	0.91	0.85	2.52
SSC	RAW	0.93	0.96	0.46	4.02
	dg2	0.95	0.97	0.38	4.87

R^2^: coefficient of determination, r: coefficient of correlation, RMSE: root mean square error, RPD: ratio performance to deviation, dg2: second derivative savitzky-golay, SSC: soluble solids content.

## 2 Experimental Design, Materials and Methods

### 2.1 Sample preparation

A total of 300 pieces sample consisting of 6 commodities belonging to the Cucurbitaceae family were prepared. Samples were harvested from gardens in the Sumedang and Bandung areas, Indonesia. The samples were cleaned of various impurities carried from the garden such as soil, leaves, and others. Data collection was done at the Horticulture Laboratory, Faculty of Agriculture, Universitas Padjadjaran.

### 2.2 Spectra data collection

Vis/NIR spectra data were obtained using the portable NirVana AG410 (Integrated Spectronics Pty, Ltd, Australia) in the wavelength range of 381-1065 nm, with a resolution of 3 nm. Each sample was irradiated 6 times, then averaged. Spectra data is automatically recorded in the spectrometer's internal memory in the form of * .isd file format. Then the spectra data is transferred to The Unscrambler X software for spectra preprocessing and model development.

### 2.3 Water content and SSC determination

The next stage after the spectra measurement is complete, namely water content and SSC determination. Each fruit is cut crosswise according to the area irradiated by the Vis/NIR spectrometer detector. The SSC of the fruit extract was evaluated in %Brix using a digital refractometer (Atago, Japan) and represents the mean of three measurements taken in each sample [3]. In the water content assessment, a sample of 30 grams was cut into small pieces and put in a small glass container and dried using an oven at 60 °C [4]. After all samples achieve a constant weight, the water content is measured. The water content is determined by calculating the weight loss after drying to the initial weight.

### 2.4 Spectra preprocessing and model development

Absorbance spectra (raw) usually contain noise which can reduce the accuracy of the interpretation of the spectra data. Therefore, spectra preprocessing is needed to improve the spectra data prior to the development stage of the calibration model [5]. The use of preprocessing spectra such as the second derivative savitzky-golay is effective in clarifying peaks and valleys in the original (raw) spectra. Calibration modeling is carried out using the principal component regression (PCR) method. Basically, PCR works by changing the spectra data into new uncorrelated variables, then performing linear regression on these new variables and the reference data. The output of this PCR is a model that can be used to predict the desired quality parameters.

## CRediT authorship contribution statement

**Kusumiyati Kusumiyati:** Visualization, Formal analysis, Supervision, Writing – original draft. **Yuda Hadiwijaya:** Writing – original draft, Formal analysis. **Ine Elisa Putri:** Writing – original draft, Formal analysis. **Agus Arip Munawar:** Writing – original draft, Formal analysis.

## Declaration of Competing Interest

The authors declare that they have no known competing financial interests or personal relationships which have or could be perceived to have influenced the work reported in this article.

## Appendix Supplementary materials

Image, application 1
